# A novel role for endothelial tetrahydrobiopterin in mitochondrial redox balance

**DOI:** 10.1016/j.freeradbiomed.2017.01.012

**Published:** 2017-03

**Authors:** Jade Bailey, Andrew Shaw, Roman Fischer, Brent J. Ryan, Benedikt M. Kessler, James McCullagh, Richard Wade-Martins, Keith M. Channon, Mark J. Crabtree

**Affiliations:** a BHF Centre of Research Excellence, Division of Cardiovascular Medicine, Radcliffe Department of Medicine, John Radcliffe Hospital, University of Oxford, Oxford OX3 9DU, United Kingdom; b Target Discovery Institute, Nuffield Department of Medicine, University of Oxford, Roosevelt Drive, Oxford OX3 7FZ, United Kingdom; c Oxford Parkinson's Disease Centre, Department of Physiology, Anatomy and Genetics, South Parks Road, Oxford OX1 3QX, United Kingdom; d Department of Chemistry, University of Oxford, South Parks Road, Oxford OX1 3QR, United Kingdom

**Keywords:** Tetrahydrobiopterin, Redox state, Nitric oxide synthase, Mitochondria, Superoxide

## Abstract

The redox co-factor tetrahydrobiopterin (BH_4_) regulates nitric oxide (NO) and reactive oxygen species (ROS) production by endothelial NOS (eNOS) and is an important redox-dependent signalling molecule in the endothelium. Loss of endothelial BH_4_ is observed in cardiovascular disease (CVD) states and results in decreased NO and increased superoxide (O_2_^-^) generation via eNOS uncoupling. Genetic mouse models of augmented endothelial BH_4_ synthesis have shown proof of concept that endothelial BH_4_ can alter CVD pathogenesis. However, clinical trials of BH_4_ therapy in vascular disease have been limited by systemic oxidation, highlighting the need to explore the wider roles of BH_4_ to find novel therapeutic targets. In this study, we aimed to elucidate the effects of BH_4_ deficiency on mitochondrial function and bioenergetics using targeted knockdown of the BH_4_ synthetic enzyme, GTP Cyclohydrolase I (GTPCH). Knockdown of GTPCH by >90% led to marked loss of cellular BH_4_ and a striking induction of O_2_^-^ generation in the mitochondria of murine endothelial cells. This effect was likewise observed in BH_4_-depleted fibroblasts devoid of NOS, indicating a novel NOS-independent role for BH_4_ in mitochondrial redox signalling. Moreover, this BH_4_-dependent, mitochondria-derived ROS further oxidised mitochondrial BH_4_, concomitant with changes in the thioredoxin and glutathione antioxidant pathways. These changes were accompanied by a modest increase in mitochondrial size, mildly attenuated basal respiratory function, and marked changes in the mitochondrial proteome and cellular metabolome, including the accumulation of the TCA intermediate succinate. Taken together, these data reveal a novel NOS-independent role for BH_4_ in the regulation of mitochondrial redox signalling and bioenergetic metabolism.

## Highlights

• BH4 deficiency results in ROS production from the mitochondria.
• This ROS production is predominantly NOS independent.
• Decreased levels of BH4 induced changes in the mitochondria, proteome, and metabolome.
• This study is the first to outline these novel roles for BH4 in determining mitochondrial redox state in endothelial cells.

## 1 Introduction

Tetrahydrobiopterin (BH_4_) is a redox-active cofactor and has long been known to be a critical regulator of nitric oxide synthase (NOS) coupling and the production of nitric oxide (NO) [1], [2]. Loss of endothelial BH_4_, via decreased synthesis or oxidation to 7,8-dihydrobiopterin (BH_2_), is associated with endothelial NOS (eNOS) uncoupling, whereby the superoxide radical (O_2_^-^), rather than NO, is produced [3], [4]. Moreover, the reaction of O_2_^-^ and NO forms peroxynitrite (ONOO^-^), leading to further loss of NO and increased uncoupling due to BH_4_ oxidation, thereby profoundly altering the balance between production of NO and reactive species (termed NO-redox balance) and associated signalling pathways [5], [6], [7]. The cellular NO-redox state is fundamental to the maintenance of vascular homeostasis and uncoupled eNOS is strongly associated with the development and progression of cardiovascular vascular disease (CVD) [8]. However, BH_4_ also has other cofactor and antioxidant roles, throughout a wide range of biological processes and pathological states, including phenylalanine catabolism, synthesis of neurotransmitters, and ether lipid metabolism [8]. Furthermore, BH_4_ has other important roles in cellular redox-sensitive signalling pathways [9], [10].

GTP cyclohydrolase I (GTPCH), encoded by *Gch1*, is the primary rate-limiting enzyme involved in the synthesis of BH_4_. Targeted augmentation of *Gch1* expression in endothelial cells has proven sufficient to normalise endothelial function, eNOS coupling, and reactive oxygen species (ROS) production in models of diabetes and atherosclerosis [11], [12], [13]. Similarly, targeted knockout of *Gch1* in endothelial cells has led to elevated blood pressure in mouse models *in vivo* [14]. However, clinical trials of BH_4_ therapy in vascular disease have been limited by systemic oxidation and limited endothelial uptake of BH_4_, highlighting the need explore the wider roles of BH_4_ in order to identify novel therapeutic targets.

Redox signalling is the specific, usually reversible, reduction/oxidation modification of signalling pathway components by reactive species and is increasingly acknowledged to be of central importance to many physiological and pathophysiological processes. Increased production of ROS is associated with a variety of disease states such as cancer [15], Parkinson's [16], and cardiovascular disease [17]. Indeed, the NO-redox balance is crucial to the maintenance of cellular homeostasis and perturbations to this balance have far-reaching effects [8]. Whilst the consequences of uncoupled NOS on O_2_^-^ have been described [18], the effects of BH_4_ deficiency on other redox pathways remain unknown. Mitochondria are major sources of cellular ROS, via the production of O_2_^-^ at various sites along the electron transport chain, and present ideal targets for therapeutic intervention.

However, therapeutic strategies to alleviate ROS production, such as through the administration of antioxidants, have proved largely unsuccessful and closer investigation of the signalling mechanisms and the loci-dependent effects of ROS production is required in order to therapeutically target specific pathways. We have previously shown that decreased levels of endothelial BH_4_ in both cell culture and animal models leads to increased cellular generation of O_2_^-^ and that a proportion of this superoxide is produced directly from uncoupled eNOS [10]. Although our previous studies have focused on this eNOS-derived fraction of cellular O_2_^-^ production in response to deficient levels of BH_4_, the major source of the increased superoxide levels has so far remained unexplored. The aim of the current study was to explore the impact of BH_4_ deficiency on redox signalling using siRNA-knockdown of the BH_4_ synthetic enzyme GTPCH in murine endothelial cells.

## 2 Results

### 2.1 Characterisation of *Gch1* knockdown-induced BH_4_ deficiency

**Fig. 1:**
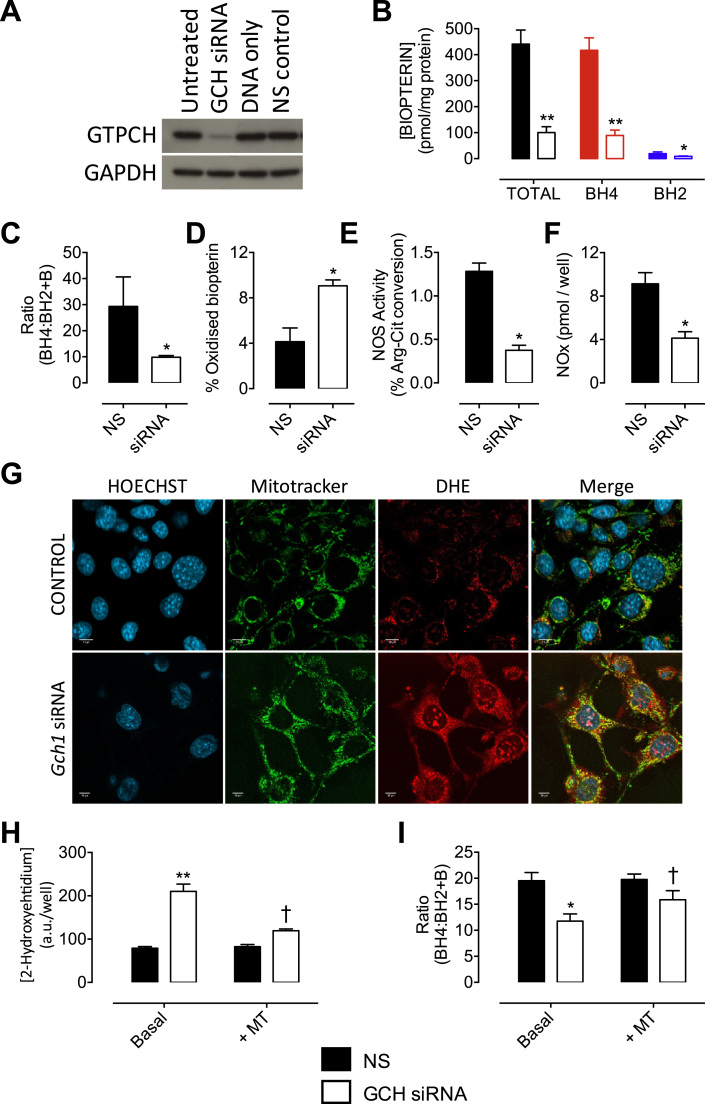
**Model of endothelial BH**_**4**_**deficiency through knock down of GTPCH leads to increased mitochondrial superoxide production**. sEnd.1 murine endothelial cells were transfected with an siRNA pool targeted to *Gch1* (siRNA) or a non-targeting (NS) scrambled control siRNA. The cells were then harvested and analysed for GTPCH protein expression by Western blotting, or biopterin levels using HPLC with electrochemical and fluorescent detection. *A)* Western blot analysis shows that cellular GTPCH protein was diminished by over 90% following exposure to *Gch1*-specific siRNA, compared to untreated cells, non-specific (NS) siRNA, and DNA only transfected control cells. *B) Gch1*-specific siRNA significantly decreased the detectable levels of cellular biopterins and *C)* the ratio of BH_4_ to BH_2_ + B, and *D)* increased the proportion of oxidised biopterins compared with NS siRNA controls. *E) Gch1* siRNA lead to significantly decreased NOS activity and *F)* NOx accumulation using a NO Analyser. n =3 (*, p<0.05) (**, p<0.01). A combination of methods was used to elucidate the source of superoxide induced by modified redox state in response to BH_4_ deficiency. *G)* sEnd.1 endothelial cells were transfected with *Gch1*-specific and control siRNA, incubated for 72 h and then exposed to MitoSOX™ Red, MitoTracker® Green and/or Hoechst nuclear stain or dihydroethidium (DHE). Confocal microscopy shows an increase in mitochondria-derived superoxide from BH_4_-deficient cells exhibited by increased MitoSOX™ fluorescence and MitoTracker® Green co-localisation. *H)* Quantitative measurements were obtained following exposure of these GCH-specific and NS control cells to dihydroethidium. The specific product, 2-hydroxyethidium, was measured by HPLC as outlined in the *‘Experimental Procedures’*; increased accumulation of 2-hydroyethidium, indicative of superoxide formation, was observed in cells with diminished levels of BH_4_. This signal was attenuated by pre-treatment with MitoTEMPO. *I)* MitoTEMPO also partially restores the attenuated BH_4_:BH_2_+B ratio following transfection of *Gch1*-specific siRNA. n =4 (*, p<0.05) (**, p<0.01) (†, p<0.05).

We first tested the effect of targeting *Gch1* gene expression, through RNA interference, on the knockdown of GTPCH protein and subsequent effects on cellular BH_4_ levels. Transfection of sEnd.1 cells with *Gch1*-specific siRNA lead to a substantial decrease in GTPCH protein, as detected by Western blotting (Fig. 1A). The depletion of GTPCH was paralleled by a ~80% decrease in cellular BH_4_ levels (Fig. 1B). The alteration of eNOS:BH_4_ ratio (previously demonstrated by our laboratory [18]), by depletion of BH_4_, also lead to a significantly decreased ratio of BH_4_ relative to oxidised biopterin species (BH_4_:BH_2_+B) and a 2-fold increase in the accumulation of oxidised biopterin species in *Gch1*-knockdown vs. NS control cells (Fig. 1C and D). BH_4_ depletion was also sufficient to significantly lower eNOS activity and NOx accumulation (Fig. 1E and F).

### 2.2 BH_4_-dependent alterations in cellular NO:redox balance determine mitochondrial redox state

Subsequent to the observation that GTPCH knockdown led to the oxidation of intracellular BH_4_, the origin of this oxidative stress was investigated using microscopic and analytical techniques. Confocal imaging of BH_4_-deficient sEnd.1 cells treated with MitoSOX™ Red demonstrated a substantial increase in mitochondrial O_2_^-^, which co-localised with the mitochondrial probe MitoTracker® Green (Fig. 1G). For quantification, we used HPLC to measure the accumulation of 2-hydroxyethidium in cells incubated with dihydroethidium, as an indicator of O_2_^-^ production. *Gch1* knockdown and GTPCH depletion resulted in a marked increase in measurable 2-hydroxyethidium (Fig. 1H) with consequent accumulation of BH_2_ and decrease in BH_4_:BH_2_ ratio (Fig. 1I). Moreover, increased ROS generation, and the subsequent effects on BH_4_:BH_2_ ratio, were both prevented by the mitochondrial-targeted antioxidant MitoTEMPO (Fig. 1H and I).

**Fig. 2:**
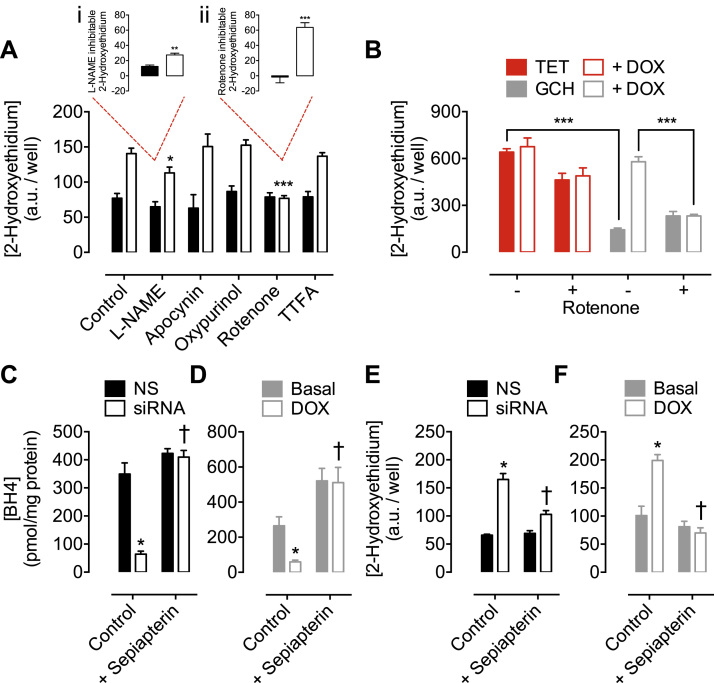
**Elevated superoxide production is attenuated by rotenone, or supplementation with sepiapterin**. *Gch1*-specific siRNA and NS treated control were incubated with DHE, and the accumulation of 2-hydoxyethidium was measured by HPLC with fluorescent detection. *A)* Pre-treatment of cells with L-NAME (200 µmol/litre), apocynin (100 µmol/litre), oxypurinol (100 µmol/litre), rotenone (2 µmol/litre) or TTFA (5 µmol/litre) reveals a significant attenuation of superoxide in BH4 deficient *Gch1*-siRNA cells treated with L-NAME (*p<0.05) or rotenone (***, p<0.001), compared to control cells. *(Inset: i, L-NAME and ii, Rotenone inhibitable fractions) B)* Similarly, GCH-tet cells demonstrate decreased 2-hydroxyethidium accumulation compares to TET cells that do not express GCH (***p<0.001). The increase in superoxide levels in GCH-tet cells following 10 days exposure to doxycycline is also significantly reduced by rotenone treatment (***p<0.001). *C and D)* Treatment of *Gch1*-siRNA and GCH-tet cells with sepiapterin restored BH_4_ levels to at least basal levels, and *E and F)* abolished the superoxide production that was elevated by BH_4_ depletion. n =4/6 (*, p<0.05) (***, p<0.001) (†, p<0.01).

To further identify the source of BH_4_-dependent ROS production, we systematically inhibited enzyme systems known to generate O_2_^-^. Selective inhibition of NADPH oxidase (by apocynin), xanthine oxidase (by oxypurinol) and mitochondrial complex II (by TTFA), had no effect on superoxide production, whilst the NOS inhibitor L-NAME resulted in only a partial decrease in O_2_^-^ levels (Fig. 2A). However, rotenone (an inhibitor of mitochondrial complex I) had a striking inhibitory effect that normalised the levels of O_2_^-^ in BH_4_-depleted sEnd.1 cells to that of control cells (Fig. 2A). This rotenone-inhibitable portion of detectable O_2_^-^ was 2.5-fold greater than that which was attenuated using L-NAME *(inset, i and ii).* This rotenone-dependent inhibition was equally apparent in BH_4_-deficient (non-eNOS expressing) GCH cells exposed to doxycycline (Fig. 2B). Furthermore, supplementation of both siRNA and Tet-Off models with sepiapterin restored BH_4_ levels (Fig. 2C and D) and diminished levels of O_2_^-^ to those of control cells (Fig. 2E and F).

**Fig. 3:**
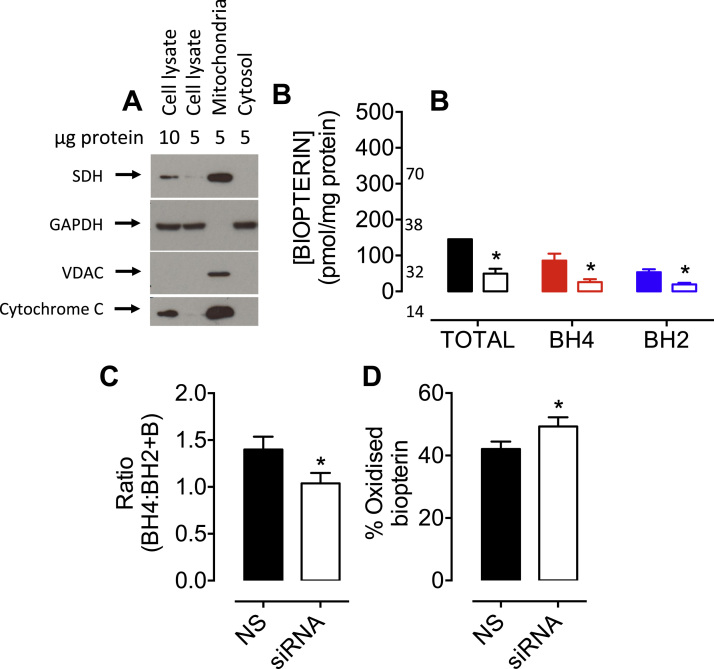
**Cellular BH**_**4**_**deficiency alters mitochondrial biopterin balance**. *A)* Enriched/isolated fractions of mitochondria were obtained from sEnd.1 cells as outlined in *‘Experimental Procedures’*, and their purity confirmed by Western blot analysis using anti-SDH, -VDAC, -cytochrome C, and -GAPDH antibodies. Importantly, these proteins were observed in the mitochondrial fraction, and GAPDH in the cytosolic-, without contamination of the mitochondrial fraction. *B)* Mitochondrial biopterin levels were determined using HPLC with electrochemical and fluorescent detectors as previously. BH_4_, BH_2_ and biopterin levels were all significantly attenuated in mitochondria isolated from BH_4_ deficient vs. NS control cells, together with *C)* a lower BH_4_:BH_2_+B ratio, and *D)* a higher percentage of oxidised biopterins (BH_2_+B). n =3 (*, p<0.05).

The effect of BH_4_ deficiency on the redox balance of isolated mitochondria was investigated. Mitochondrial preparations were assessed by Western blotting; anti-succinate dehydrogenase (SDH), -voltage-dependent anion channel (VDAC), and -cytochrome C antibodies confirmed mitochondrial enrichment, without cytosolic GAPDH contamination (Fig. 3A). The total amount of biopterins detected in mitochondria from sEnd.1 endothelial cells was 3-fold lower in *Gch1*-specific siRNA-treated cells (49.7±13.6 vs 145.5±28.7 pmol/mg protein), compared to mitochondria isolated from non-specific siRNA-treated control cells. Mitochondria from BH_4_-replete control cells contained 85.6±19.5 pmol/mg protein of BH_4_ vs. 26.0±8.0 pmol/mg protein in *Gch1-*siRNA cells, and 53.6±8.3 vs 19.5±4.3 pmol/mg protein of BH_2_ (Fig. 3B). These data imply an oxidising mitochondrial environment, as the BH_4_:BH_2_ ratio is much lower in the mitochondria compared to that in whole cells (29.3±11.3 in whole cells *vs* 1.4±0.14 in mitochondria). The lower mitochondrial BH_4_:BH_2_ ratio was even more perturbed by the changes in redox balance induced by diminished BH_4_ levels—the ratio of BH_4_:BH_2_ being significantly decreased in mitochondria from *Gch1*-specific siRNA-treated cells *versus* non-specific controls (1.04±0.11 vs 1.4±0.14), with the relative amount of oxidised biopterins increasing (Fig. 3C and D; *P<0.05).

### 2.3 Diminished BH_4_ levels and subsequent alterations in redox state alter mitochondrial size and function

**Fig. 4:**
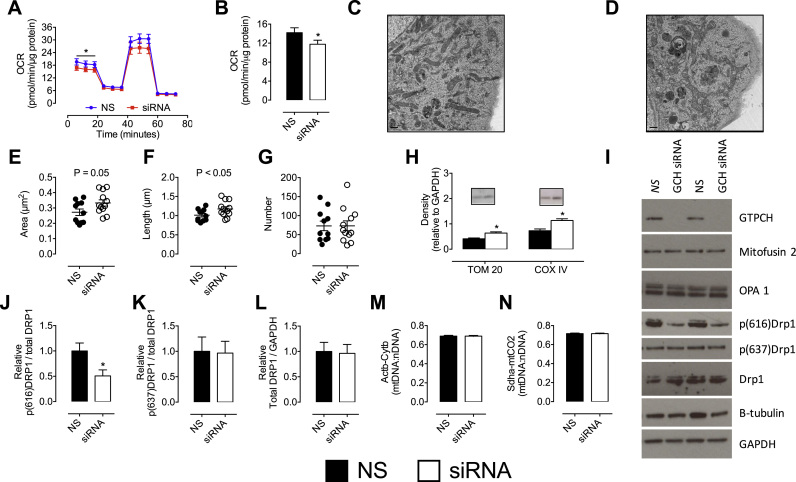
**Altered mitochondrial redox state induces changes in mitochondrial structure and function**. *A and B)* The observed changes in mitochondrial redox state also altered mitochondrial bioenergetics, as shown by the significant decrease in basal oxygen consumption rates (OCR) in *Gch1*-specific siRNA transfected cells compared to their NS treated control cells. OCR was measured using an XFe96 extracellular flux assay. OCR measurements were taken at baseline and then following sequential injections of oligomycin, FCCP and rotenone+antimycin A OCR was significantly decrease in siRNA-treated (red) cells at baseline (vs control, blue). Analysis of electron micrographs from *C)* NS control cells, and *D) Gch1* siRNA transfected cells reveals that cellular mitochondria exhibit *E)* a trend towards increased area, *F)* and a significant increase in length, *G)* independent of a difference in absolute number (averages from NS =11 cells, *Gch1* =12 cells). *H)* Evidence for increased mitochondrial size and mitochondrial fusion was supported by Western blot analysis; TOM20 and COXVI increased in *Gch1* siRNA cells compared to NS control cells. *I-L)* Further Western blotting demonstrates a striking decrease in p(616)Drp1, but not p(637)Drp1, with no difference in mitofusin 2 or OPA1. *M and N)* No change in the quantity of mtDNA was observed between GCH siRNA and control cells. All data represents 6–8 independent experiments (*, p<0.05). n =4–5 for Western blots.

To examine further the impact of BH_4_ depletion on mitochondrial function, mitochondrial respiration, morphology, and protein composition were investigated. Mitochondrial OCR was assessed under basal conditions, followed by consecutive measurements after exposure to oligomycin, Carbonyl cyanide-*4*-(trifluoromethoxy)phenylhydrazone (FCCP), and antimycin A/rotenone compounds that variously inhibit or accelerate the electron transport chain. Determination of basal respiration showed a small but significant decrease in sEnd.1 cells deficient in BH_4_ (Fig. 4A and B), independent of any changes in spare respiratory capacity, proton leak, or ATP linked respiration (data not shown).

Treatment of endothelial cells with *Gch1*-specific siRNA, and the subsequent BH_4_ deficiency, resulted in modest changes in mitochondrial size (measured by electron microscopy, shown in Fig. 4C and D) and levels of certain important proteins relating to mitochondrial content/dynamics. Mitochondria from BH_4_-deficient endothelial cells showed increased length and area compared to control, in the absence of any change in absolute mitochondrial number (Figs. 4E-G; *P<0.05). Western blots also supported evidence of an increase in mitochondrial size, with TOM20 and COXIV (inner and outer membrane markers, respectively) increased in BH_4_-deficient mitochondria (Fig. 4H). Furthermore, the phosphorylation status of the master fission regulatory protein, dynamin related protein-1 (DRP1) was significantly decreased at the activating phosphorylation site, serine 616 (Fig. 4J) without concurrent changes at the inhibitory site of serine 637 (Fig. 4K). These changes were independent of alterations in the amount of total DRP1 protein (Fig. 4L) in control *Gch1* siRNA cells (Fig. 4I). Markers of mitochondrial fusion (OPA1, and Mitofusin 2) (Fig. 4I) and mitochondrial DNA content (Fig. 4M and N) showed no difference between control and *Gch1*-specific siRNA treatments, as measured by Western blotting (Supplementary Fig. 2).

### 2.4 BH_4_ is a redox-dependent modulator of mitochondrial signalling

**Fig. 5:**
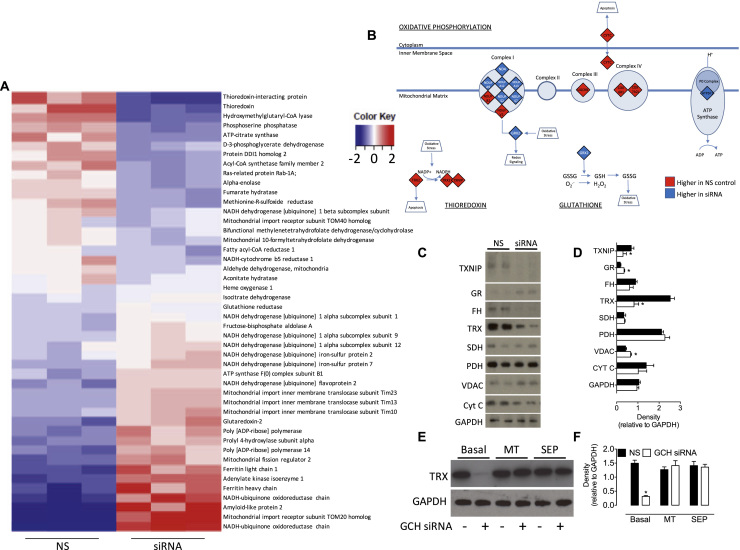
**Modulation of the mitochondrial proteome by BH**_**4**_. *Gch1* siRNA and control cells underwent proteomic analysis by mass spectrometry. *A)* Normalised relative abundance of detected proteins was determined as described in the *‘Experimental Procedur*es’ (data shown in Supplemental Tables 1 and 2), and significant changes in 46 proteins presented in the heat map. Data presented represent 46 candidate proteins, changed by over 1.5-fold and 1% false discovery rate. Proteins were ranked by fold difference between *Gch1* siRNA and NS expressing cells. Higher abundant proteins are expressed in Red, and those of lower abundance, compared to the average of all values, are shown in Blue. *B)* Ingenuity Pathway Analysis was used to highlight which pathways where experimentally modulated by BH_4_ in our cell model. Thioredoxin (downregulated) and glutathione (upregulated) antioxidant systems were found to be down altered in BH_4_ deficient cells, as well as several proteins from the ‘Oxidative Phosphorylation’ pathway identified by IPA. *C)* These proteomic data were confirmed by Western blot analysis, and those ‘hits’ shown to be modified in abundance to the largest extent were validated using specific antibodies. TXNIP, TRX and FH were shown to be decreased upon knockdown of *Gch1*, and GR and VDAC were demonstrated to be significantly elevated. *D)* Densitometric analysis of Western blot bands. *E) and F)* Exposure of NS or *Gch1*-siRNA treated endothelial cells to either MitoTEMPO or sepiapterin was sufficient to restore the decreased levels of a candidate protein, TRX, induced by BH_4_ deficiency. n =3 (*, P<0.05).

**Fig. 6:**
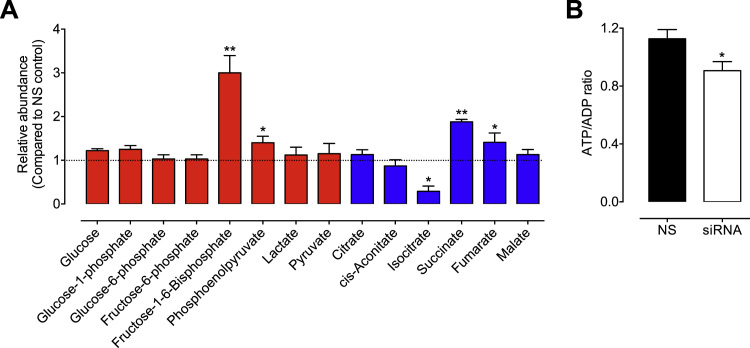
**Metabolomic analysis reveals a role for BH**_**4**_**in metabolism**. sEnd.1 endothelial cells were transfected with *Gch1*-specific siRNA and compared to cells transfected with NS scrambled control siRNA. Cell lysates were harvested and underwent metabolomics analysis as outlined in the *‘Experimental Procedures’. A)* Glycolysis (red) and TCA cycle (blue) metabolites were detected and quantification established by comparison with known standards. Succinate, fumarate, phosphoenolpyruvate and Fructose 1-,6-bisphosphate were significantly increased in response to *Gch1*-specific siRNA transfection, while isocitrate levels were sown to be markedly diminished compared to NS control cells. *B)* The impact of these changes in mitochondrial electron transport chain protein expression, glycolytic and TCA cycle metabolites, was to significantly decrease ATP/ADP ratio induced following knockdown of *Gch1* by targeted siRNA, and the resulting BH_4_ deficiency. n =3 (*, p<0.05) (**, p<0.01) (***, p<0.001).

The effects of BH_4_-dependent perturbations in mitochondrial redox balance on both the mitochondrial proteome and cellular metabolome were in investigated using mass spectrometry. Analysis of the mitochondrial proteome showed that BH_4_ deficiency resulted in proteome remodelling, as illustrated by the heat map in Fig. 5A. BH_4_ deficiency led to profound changes in the abundance of proteins involved with mitochondrial dynamics redox signalling, and bioenergetic metabolism. For example, proteins with the most significantly altered response to reduced levels of BH_4_ and subsequent altered redox homeostasis included those involved in antioxidant signalling mechanisms; thioredoxin (TRX) and thioredoxin-interacting protein (TXNIP) both decreased in abundance in *Gch1*-specific siRNA endothelial cells vs control cells, whilst glutathione reductase (GR) and glutaredoxin-2 (GRX2) were both increased. Metabolic proteins fumarate hydratase and isocitrate dehydrogenase also significantly decreased in response to BH_4_ deficiency. Moreover, mitochondrial import proteins (TOM20, TIM10, TIM13 and TIM23) were upregulated (Fig. 5A). The roles and impact of these changes are outlined in the discussion. Ingenuity Pathway Analysis (IPA) then identified downstream pathways modified in sEnd.1 endothelial cells following knockdown of *Gch1* and revealed oxidative phosphorylation, thioredoxin, and glutathione as affected pathways (Fig. 5B). These data were confirmed by Western blot analysis, which also showed TXNIP, TRX, and fumarate hydratase to be decreased, while the abundance of GR was increased in *Gch1*-specific siRNA vs. non-specific siRNA treated endothelial cells (Fig. 5C and D). These changes were dependent on both cellular BH_4_ and mitochondria-derived ROS, as demonstrated by Western blotting; the decrease in TRX protein expression (a candidate protein selected from our proteomics data) was prevented following inhibition of mito-ROS by MitoTEMPO and after supplementation of intracellular BH_4_ using sepiapterin (Fig. 5E and F). The impact of these changes could also be observed at the cellular metabolite level; levels of succinate (**P<0.01) and fumarate (*P<0.05) were markedly elevated, whilst isocitrate was decreased in *Gch1*-specific siRNA vs. non-specific siRNA treated endothelial cells (*P<0.05; Fig. 6A). Similarly, the level of fructose 1,6-bisphosphate was strikingly elevated (3-fold) in BH_4_-deficient endothelial cells, compared to control siRNA treated cells (**P<0.01). The overall effect of these changes on cellular energy levels was a modest, but significant, decrease of the ATP/ADP ratio in BH_4_-deficient cells (Fig. 6B).

## 3 Discussion

In this study, we investigated BH_4_-dependent mitochondrial redox signalling using both siRNA knockdown of *Gch1* in endothelial cells, and tetracycline-regulatable *GCH1* fibroblast (“GCH”) cell-culture models of BH_4_ deficiency. *Gch1* knockdown and consequent BH_4_ deficiency had profound effects on mitochondria-derived O_2_^-^ and the mitochondrial proteome, whilst also affecting levels of several important cellular bioenergetic metabolites. These findings paralleled modest, yet consistent, changes in mitochondrial size and basal respiration, together suggesting a critical role for BH_4_ deficiency in mitochondrial redox signalling and homeostasis.

Whilst we have previously described the effects of BH_4_ deficiency on eNOS uncoupling-dependent O_2_^-^ generation in Tet-regulated GCH/eNOS and sEnd.1 endothelial cells [19], the present study highlights an important fraction of the increased O_2_^-^ production due to BH_4_ deficiency that is not inhibited by treatment with L-NAME, suggesting an eNOS-independent source. Moreover, this increase in O_2_^-^ production in BH_4_-deficient cells was normalised by either scavenging with MitoTEMPO, restoration of BH_4_ levels, or the pharmacological inhibition of mitochondrial complex I in both siRNA and Tet cell models, together implicating an eNOS-independent component of increased O_2_^-^ production that is specifically mitochondrial in origin. Additionally, O_2_^-^ production was also visibly localised to the mitochondria when imaged after incubation with DHE, thus further substantiating the finding. Whilst it is likely that O_2_^-^ derived from uncoupled eNOS can induce further ROS generation via a feed-forward mechanism [20], data from the GCH model (which inherently lacks NOS) corroborate the notion of an eNOS-independent mechanism linking BH_4_ deficiency and mitochondrial O_2_^-^ overproduction. However, the specific role of BH_4_ within the mitochondria is yet to be elucidated and complicated by uncertainties regarding the presence or absence of mitochondria-specific NOS. Recently, Sethumadhavan et al. [21] showed that increasing BH_4_ in cardiomyocytes affected redox state and mitochondrial function independent of differences in NO generation. This finding further supports our hypothesis of an NOS-independent role for BH_4_ involving the mitochondria, albeit in model overexpressing GTPCH many fold. Notwithstanding the magnitude of this overexpression, there appears to be mounting evidence for an alternate role for BH_4_ in the mitochondria, beyond its canonical function in NOS coupling. However, to our knowledge, our study is the first report of an eNOS-independent role for BH_4_ in the excess generation of mitochondrial O_2_^-^ and associated redox signalling.

Concomitant with BH_4_ deficiency and increased O_2_^-^ production were decreases in both overall BH_4_ and the ratio of BH_4_ to oxidised biopterin species (BH_2_+B) in the mitochondria, indicating that genetic manipulation of GTPCH levels has specific effects within the mitochondrial compartment. Whilst both mitochondrial biopterins and GTPCH localisation have previously been reported [22], [23], [24], the concept of GTPCH producing BH_4_ specifically within the mitochondrial compartment is one that merits more thorough investigation. Similarly, BH_4_ transport is poorly understood, although it has been suggested that cellular uptake of BH_4_ possibly occurs via either urate transporters (URAT1), organic anion transporters (OATs), or multidrug-resistance-associated proteins (MRPs) in the kidney [25]. However, the fact that sepiapterin rescues the increased ROS generation induced by BH_4_ deficiency in the mitochondria suggests that BH_4_ may be indeed be transported there and further study is required.

That we show at least partial rescue of the cellular ratio of BH_4_ to oxidised biopterins, via the addition of the superoxide-scavenging MitoTEMPO, is an indication that mitochondrial O_2_^-^ overproduction may be another important mediator of mitochondrial BH_4_ bioavailability, suggesting a vicious cycle of events associated with BH_4_ depletion and ROS overproduction. Whilst we have previously shown that the ratio of BH_4_ to oxidised biopterins is more important to eNOS coupling and NO-redox balance than BH_4_ levels *per se* [26], it remains to be determined whether this is true for any eNOS-independent roles of BH_4_ in the mitochondria.

We hypothesised that BH_4_ deficiency and concomitant increase in mitochondrial O_2_^-^ would be associated with altered bioenergetic metabolism and changes in mitochondrial respiration rates. Whilst BH_4_ depletion was associated only with a small decrease in basal OCR, there were profound changes in certain TCA metabolite levels and the cellular ATP/ADP ratio, suggesting an important bioenergetic component. The modest decrease in basal OCR observed under conditions of BH_4_ deficiency is suggestive of mild impairment of mitochondrial respiratory function; however, the uncoupled reserve capacity remained unchanged (data not shown), indicating that the overt effect of BH_4_ deficiency on mitochondrial functional respiratory capacity was limited. However, given the relatively short duration of this *in vitro* knockdown model (72 h) and the highly adaptive nature of mitochondria to ensure maintenance of functional respiratory capacity, it would be prudent to investigate the effects of long-term BH_4_ depletion on mitochondrial bioenergetics and oxygen consumption *in vivo*. Similarly, BH_4_ depletion was also associated with a modest increase in mitochondria size. Protein analysis by Western blotting suggests this is due to decreased activity of fission machinery, as evidenced by the observation that DRP1 was shown to be less phosphorylated at the activating phosphorylation site of serine 616, without any changes observed in fusion proteins (mitofusin 2 and OPA1). Nevertheless, these modest changes in size and basal oxygen utilisation paralleled profound changes in mitochondrial O_2_^-^ generation and protein import into the mitochondria, which are well equipped to adapt to metabolic stress, thus preserving vital respiratory function.

Our studies also reveal changes in important cellular metabolites that further link mitochondrial bioenergetic metabolism and BH_4_ deficiency. The accumulation of both succinate and fumarate are an interesting finding that suggests BH_4_ deficiency has an effect on key mitochondrial metabolites of the TCA cycle. Chouchani et al. [27] proposed that succinate accumulation (resulting from ischaemia) is the major driver of mitochondrial superoxide production via complex I. Indeed, it may be prudent to explore the impact of succinate accumulation on mitochondrial superoxide generation in BH_4_-deficient cells. Furthermore, the stark decrease in isocitrate, and striking increase in fructose 1,6-bisphosphate (possibly as a compensatory protective mechanism against the oxidative insult induced by BH_4_ deficiency, akin to that observed in ischemic injury [28]) further highlight a profound change in TCA and glycolytic metabolism under conditions of BH_4_ deficiency. Thus, whilst the link between BH_4_ deficiency, mitochondrial bioenergetic metabolism, and increased superoxide generation remains unclear, it warrants further investigation. Taken together, these results suggest that BH_4_ deficiency specifically alters mitochondrial metabolic/bioenergetic homeostasis, in tandem with increased mitochondrial superoxide production *in vitro*.

We also investigated the effect of BH_4_ deficiency on the cell proteome and found a dramatically altered profile, which included changes in a wide range of proteins associated with mitochondrial redox signalling, mitochondrial metabolism, antioxidant defence, and stress response. The thioredoxin and glutaredoxin systems are both critical mediators of mitochondrial redox homeostasis [29]. Both TRX and TXNIP were downregulated under conditions of BH_4_ deficiency, whilst GR and the mitochondrial GRX2 were upregulated, reflecting an altered redox balance due to increased ROS generation. This change in proteins central to major redox signalling/antioxidant pathways was confirmed by Western blot and serves to highlight differential cellular responses to BH_4_ deficiency, operating via discrete redox signalling pathways. Indeed, the thioredoxin and glutaredoxin systems are important regulators of redox signalling specifically because of their ability to both cooperate and function independently of each other [30]. Moreover, the addition of a mitochondrial superoxide scavenger (MitoTEMPO) and supplementation of BH_4_ levels using sepiapterin, are both sufficient to rescue the depletion of thioredoxin in BH_4_-deficient cells. When taken together with the data presented herein that demonstrates the ability of MitoTEMPO and sepiapterin to individually normalise the decrease in BH_4_, and the increase in ROS in *Gch1* siRNA-treated cells, this shows strong evidence that BH_4_-deficiency, and consequent mitochondrial ROS generation, negatively affect the abundance of these selected cellular enzymatic antioxidants.

Similarly, BH_4_ deficiency induced changes in various other redox proteins possessing ferroxidase and oxidoreductase activity—such as ferritin light/heavy chains and phosphoglycerate dehydrogenase that were up- and downregulated, respectively—reinforcing the importance of BH_4_ in redox signalling homeostasis. Further evidence for a redox-regulatory role for BH_4_ was provided by upstream IPA pathway analysis predicting that Nrf2-dependent signalling may be decreased in response to *Gch1* knockdown within endothelial cells (data not shown), supporting our previous findings in primary macrophages isolated from the *Gch1*^fl/fl^Tie2cre knockout mouse [9].

The mitochondrial import proteins (TOM20, TIM10, TIM13, and TIM23) were all upregulated, suggesting a redox-dependent shift in mitochondrial dynamics [31]. However, mitochondrial fission regulator 2 (Mtfr2), DRP, and OPA1, which regulate mitochondrial fusion/fission, were unchanged between groups [32]. Overall, these differences were concomitant changes observed in mitochondrial size/shape and basal respiration, and point towards perturbations in mitochondrial homeostasis induced by BH_4_ deficiency. Moreover, proteins associated with the control of bioenergetic function decreased in our BH_4_-deficienct model, including those of the TCA cycle, such as Acyl-CoA synthetase, ATP-citrate synthase, isocitrate dehydrogenase, and fumarase—possibly reflecting altered control of bioenergetic flux at specific points in the pathway. Finally, certain subunits of electron transport chain proteins were upregulated in BH_4_-deficient cells, including NADH-ubiquinone oxidoreductase chain 1 and 2 (complex I) and ATP synthase F(0) complex subunit B1 (complex V), indicating BH_4_ deficiency induced alterations in electron transport capacity. In total, these results show a striking change in the proteome of cells deficient of BH_4_ and highlight an overall change in mitochondrial dynamics, bioenergetics, and redox signalling.

**Fig. 7:**
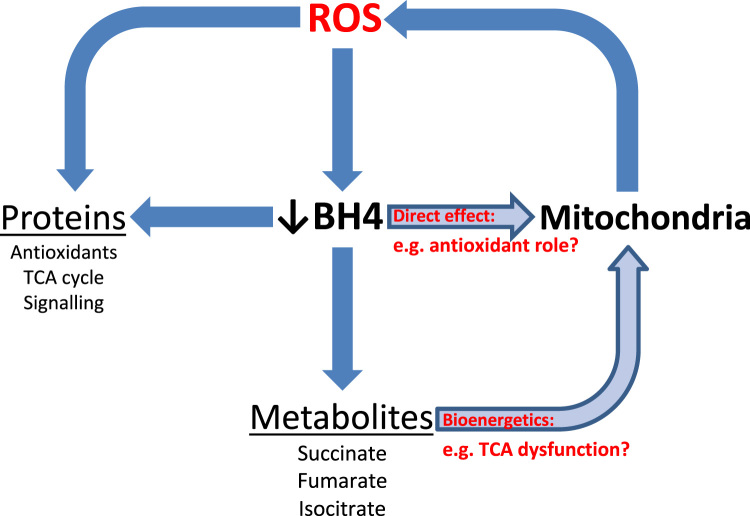
**Schematic representation of the interaction and crosstalk between BH4, ROS generation and mitochondrial signalling**. A feed forward cascade of ROS generation from the mitochondria and BH4 oxidation results in deleterious effects on mitochondrial metabolism, protein signalling and function.

In summary, this study shows increased mitochondrial O_2_^-^ in *in vitro* models of BH_4_ deficiency, therefore providing strong evidence of an eNOS-independent role for BH_4_ in mitochondrial redox signalling. BH_4_ is a redox-active molecule and oxidation to BH_2_ plays a significant part in the decreased bioavailability observed in disease states. In accordance with previous reports [22], [23], [24], we have detected biopterins in mitochondria. We also evidence lower levels of BH_4_ in the mitochondria of BH_4_-depleted cells and show that, of the remaining, a greater proportion is oxidised. It is therefore plausible that BH_4_ is having a direct effect inside mitochondria, leading to increased O_2_^-^. Mitochondria are known critical sources and targets of ROS, under both physiological and pathophysiological conditions [21], [22] and elucidating the role of BH_4_ is of vital importance. Enzymatic cofactor-independent roles of BH_4_ so far remain relatively unexplored, although a direct cofactor role in the mitochondria has previously been alluded to [24], [33]. The overlap of the interconnecting themes of BH_4_, altered redox signalling and mitochondrial bioenergetic metabolism present an opportunity to explore this concept further (Fig. 7). BH_4_ has been shown to act a scavenger of ROS *in vitro* [34]; however, this study shows effects of BH_4_ deficiency that suggest a more profound role for BH_4_ in the mitochondrial homeostasis. It is interesting to note that GTPCH acts directly on guanosine triphosphate (GTP), a product of the TCA cycle, which we have shown to be perturbed by BH_4_ deficiency. It has been shown that GTP is a regulator of BH_4_ synthesis and it would be interesting to investigate the effect of BH_4_/GTPCH deficiency on GTP production and TCA activity [35]. Indeed, perturbed TCA cycle activity, and GTP production may underlie the increase in succinate observed in the current study.

Redox imbalance, oxidative stress, and decreased NO and BH_4_ are well-established markers of endothelial dysfunction in vascular pathologies, including hypertension, diabetes and atherosclerosis. However, therapeutic strategies aimed at alleviating ROS overproduction have largely proved unsuccessful and an understanding of redox signalling mechanisms and loci-dependent causes and effects of ROS production are required. Elucidating the role of BH_4_ in the maintenance of mitochondrial redox homeostasis is fundamental to this understanding and the mitochondrion presents a prime target for therapeutic intervention.

## 4 Experimental procedures

### 4.1 Cell culture

sEnd.1 murine endothelial cells and Tet-regulatable GCH cells were cultured in DMEM (Invitrogen), supplemented with 10% FBS, glutamine (2 mmol/litre), penicillin (100 units/ml), and streptomycin (0.1 mg/ml). We used NIH 3T3 murine fibroblasts stably transfected with a *tet*-off transactivator construct. In the presence of doxycycline, binding of the transactivator is blocked, and gene expression is prevented. These initial 3T3-*tet*-off cells, previously shown to express neither eNOS nor GTPCH and also confirmed to be devoid of neuronal NOS, inducible NOS, and eNOS protein, were stably transfected with a plasmid encoding hemagglutinin (HA) antigen-tagged-human GCH1 under the control of a tetracycline-responsive element, Individual colonies were isolated and analysed for GCH1 expression and a cell line, termed “GCH cells,” was established from expansion of a single colony. GCH cells were stably transfected with a plasmid encoding a human eNOS-eGFP fusion protein and clones were picked. All of the cell lines underwent three rounds of clonal selection. GCH cells were maintained in medium containing Hygromycin B (200 μg/ml) and Geneticin® (200 μg/ml). NIH 3T3 murine fibroblasts were stably transfected with a Tet-Off transactivator construct, as previously described. Doxycycline (1 μg/ml) was added to cell culture media to abolish transcription of *Gch1* mRNA where appropriate as previously described [19].

### 4.2 Gch1 knockdown by RNA interference

*Gch1*-specific, ON-TARGETplus SMARTpool siRNA were purchased from Dharmacon Thermo Scientific. The siRNA were used as a pool of four specific siRNA duplexes with the following sequences: Duplex 1, GGUAGAAUGCUAAGUACGU; Duplex 2, CGAGAAGUGUGGCUACGUA; Duplex 3, GAGAAGGGAGAAUCGCUUU; and Duplex 4, AGUAGUGAUUGAAGCGACA. 24 h prior to transfection, sEnd.1 cells were seeded into 6-well plates. Cells were then transfected with *Gch1*-specific siRNA (100 nmol/litre), or nonspecific (NS) pooled duplex negative control siRNA (100 nmol/litre), using DharmaFect1 transfection reagent (Dharmacon). Cells were cultured for 72 h and gene silencing was detected by analysis of GTPCH protein expression by means of Western blotting using GTPCH-specific antibodies.

### 4.3 Biopterin quantification by HPLC with electrochemical detection

BH_4_, BH_2_, and biopterin levels in cell and mitochondrial lysates were determined by HPLC followed by electrochemical and fluorescent detection, as described previously [36], [37]. Briefly, the cells were grown to confluency and harvested by trypsinisation. Mitochondria from confluent T175 flasks were isolated using the Qproteome Mitochondrial Isolation Kit (Qiagen). Sample pellets were resuspended in PBS (50 mmol/litre), pH 7.4, containing dithioerythritol (1 mmol/litre) and EDTA (100 μmol/litre) and subjected to three freeze-thaw cycles. Following centrifugation (15 min at 17,000*g*, 4 °C), the samples were transferred to new, cooled microtubes and precipitated with ice-cold extraction buffer containing phosphoric acid (1 mol/litre), trichloroacetic acid (2 mol/litre), and dithioerythritol (1 mmol/litre). The samples were vigorously mixed and then centrifuged for 15 min at 17,000*g*, 4 °C. The samples were injected onto an isocratic HPLC system and quantified using sequential electrochemical (Coulochem III, ESA Inc.) and fluorescence (Jasco) detection. HPLC separation was performed using a 250 mm, ACE C-18 column (Hichrom) and mobile phase comprising of sodium acetate (50 mmol/litre), citric acid (5 mmol/litre), EDTA (48 μmol/litre), and dithioerythritol (160 μmol/litre) (pH 5.2) (all ultrapure electrochemical HPLC grade), at a flow rate of 1.3 ml/min. Background currents of +500 μA and −50 μA were used for the detection of BH_4_ on electrochemical cells E1 and E2, respectively. 7,8-BH_2_ and biopterin were measured using a Jasco FP2020 fluorescence detector. Quantification of BH_4_, BH_2_, and biopterin was made by comparison with authentic external standards and normalised to sample protein content.

### 4.4 Quantification of superoxide production by HPLC

Measurement of 2-hydroxyethidium formation by HPLC was used to quantify O_2_^-^ production, from methods adapted from those described previously [38], [39]. Cells grown in 6-well plates at 70–80% confluency were incubated in the presence or absence of L-NAME (200 μmol/litre), rotenone (2 μmol/litre), apocynin (100 μmol/litre), TTFA (5 μmol/litre), oxypurinol (100 μmol/litre), and sepiapterin (10 μmol/litre). After 30 min, dihydroethidium (25 μmol/litre) was added and the cells were then incubated for a further 20 min, at 37 °C. Cells were then harvested by scraping and centrifuged, before being lysed in ice-cold methanol. Hydrochloric acid (100 mmol/litre) was added (1:1 v/v) prior to loading into the autosampler for analysis. All samples were stored in darkened tubes and protected from light at all times. Separation of dihydroethidium, 2-hydroxyethidium, and ethidium was performed using a gradient HPLC system (Jasco) with an ODS3 reverse phase column (250 mm, 4.5 mm; Hichrom) and quantified using a fluorescence detector set at 510 nm (excitation) and 595 nm (emission). A linear gradient was applied from Mobile phase A (0.1% trifluoroacetic acid) to Mobile phase B (0.085% trifluoroacetic acid in acetonitrile), over 23 min (30–50% acetonitrile).

### 4.5 Imaging mitochondrial superoxide production

Cells grown on confocal dishes were washed once in Krebs-HEPES buffer, incubated with MitoTracker Green (Thermo Fisher Scientific), before exposure to MitoSOX™ Red (Thermo Fisher Scientific) for 20 min at 37 °C. Hoechst nuclear stain was added 5 min before imagining with a Zeiss 510 Metahead confocal microscope, using excitation and emission wavelengths of 488 nm and 595 nm, respectively.

### 4.6 Mitochondria isolation

Cells grown to confluency in T175 flasks were harvested by trypsinisation and pelleted at 400*g*, at 4 °C. Mitochondria were isolated using the Qproteome Mitochondria Isolation Kit (Qiagen), as per the manufacturer's instructions. All steps were carried out on ice or at 4 °C and mitochondrial pellets were stored at −80 °C for BH_4_, Western, or proteomic analysis.

### 4.7 Western blotting

Cell or mitochondrial lysates were prepared by homogenisation in ice-cold CelLytic™ M buffer (Sigma) containing protease inhibitor cocktail (Roche Applied Science). Lysates were centrifuged at 17,000*g* for 10 min at 4 °C, and samples were prepared using LDS sample buffer (Invitrogen). Western blotting was carried out using standard techniques with anti-GAPDH, -VDAC, -cytochrome C, -SDH, –GTPCH, -TXNIP, -GR, -FH, -TRX, -PDH, -TOM20, -COXIV, -Mitofusin 2, -OPA1, DRP1, p(ser616)DRP1, p(ser637)DRP1 and β-tubulin antibodies.

### 4.8 Proteomics

Mitochondrial pellets were dissolved in 6 M urea in 50 mM ammonium bicarbonate. Dithiothreitol (10 mM) was added and samples were incubated at 60 °C for 45 min. Samples were diluted 1:10 in 50 mM ammonium bicarbonate and sequencing grade modified trypsin (Promega) was added (1:50 w/w) for overnight digestion. PMSF (0.5 mM) stopped the reaction and samples were dried down in a vacuum concentrator (Speedvac, Thermo Scientific) and resuspended in acetonitrile (2%) and TFA (0.1%). Samples were desalted using ZipTipC18 pipette tips (Millipore), vacuum concentrated and reconstituted in acetonitrile (2%) and TFA (0.1%), before mass spectrometric analysis. LC-MS/MS was performed using a Q Exactive mass spectrometer, coupled with a Dionex Ultimate 3000 UPLC. Samples underwent online desalting using a Trap column (PepMAP C18, 300 µm×5 mm, 5 µm particle, Thermo). Subsequently, a nEASY column (PepMAP C18, 75 µm×500 mm, 2 µm particle, Thermo) was used with a flow rate of 250 nl/min over 60 min and a gradient of 2–35% acetonitrile in 5% DMSO /0.1% Formic acid to achieve UPLC separation.

Survey scans were acquired with a resolution of 70,000 at 200 *m/z* and an ion target of 3E6 between 380 and 1800 *m/z* for up to 100 ms. MS/MS spectra were acquired with a resolution of 17,500 and an ion target of 1E5 for up to 128 ms and a fixed first mass of 100 *m/z* and normalised collision energy of 28. Selected precursors were picked above a threshold of 4.7E7 counts and excluded for 27 s. Experiments were conducted in biological triplicates and all sample analysed in a single sample batch.

### 4.9 Proteomic data analysis

For label free quantitation raw data were imported into Progenesis QI (Waters) with default settings. MS2 spectra were identified with Mascot v 2.5 (Matrix Science) by searching Uniprot/Swissprot (retrieved 12/08/2014) with taxonomy *Mus musculus,* using 10 ppm and 0.05 Da peptide mass/fragment mass tolerances. Allowance was made for 1 missed cleavage site. Carbamidomethylation (C) was selected as fixed modification and Deamidation (N/Q) and Oxidation (M) as variable modifications. Data was exported for integration in Progenesis with a peptides false discovery rate of 1%. Furthermore, spectra with a peptide score <20 were discarded. Peptide abundances were mean normalised to identified features only.

### 4.10 Metabolomics

Metabolite extraction: sEnd.1 endothelial cells transfected with *Gch1*-specific siRNA were compared to cells transfected with NS scrambled control siRNA. Briefly, 200uL of 80% ice-cold, aqueous MeOH was added to the cell pellet containing approximately 1 million (pre-counted) frozen cells. The cell pellet was disrupted through aspiration to obtain a cell suspension in 80% MeOH. The cell suspension was then clarified by centrifugation at 14,800 rpm for 20 min at 4 °C. The supernatant was removed and placed in an autosampler vial and kept at −80 °C. On the day of analysis, the extract was allowed to warm to 4 °C in the chilled autosampler and then injected directly onto the system.

Metabolite analyses were performed using a Thermo Scientific ICS-5000+ ion chromatography system coupled directly to a Q-Exactive HF Hybrid Quadrupole-Orbitrap mass spectrometer with a HESI II electrospray ionisation source (Thermo Scientific, San Jose, CA). The ICS-5000+ HPLC system incorporated an electrolytic anion generator (KOH) programmed to produce an OH^-^ gradient over 37 min. An inline electrolytic suppressor removed the OH^-^ ions and cations from the post-column eluent prior to MS analysis (Thermo Scientific Dionex AERS 500). A 10 μL partial loop injection was used for all analyses and the separation was performed using a Thermo Scientific Dionex IonPac AS11-HC 2×250 mm, 4 µm particle size column with a Dionex Ionpac AG11-HC 4 µm 2×50 guard column inline. The IC flow rate was 0.250 ml/min. The total run time was 37 min and the hydroxide ion gradient comprised as follows: 0 mins, 0 mM; 1 min, 0 mM; 15 mins, 60 mM; 25 mins, 100 mM; 30 mins, 100 mM; 30.1 mins, 0 mM; 37 mins, 0 mM. Analysis was performed in negative ion mode using ascan range from 80 to 900 and resolution set to 70,000. The tune file source parameters were set as follows: sheath gas flow 60; aux gas flow 20; spray voltage 3.6; capillary temperature 320; S-lens RF value 70; heater temperature 450. AGC target was set to 1E6 and the Max IT value was 250 ms. The column temperature was kept at 30 °C throughout the experiment. Full scan data were acquired in continuum mode. Peaks retention times were identified from the injection of authentic standards and peaks from unknown samples were identified using a combination of accurate mass analysis (<2 ppm) and retention time using Thermo Scientific Quanbrowser software (Thermofisher Scientific, Hemmel, UK).

### 4.11 Seahorse XF^e^96 OCR analysis

48–72 h post siRNA transfection, sEnd.1 cells were plated at a density of 7.5×10^4^ cells per well into XF^e^96 microplates. Cells were left to attach for 24 h before extracellular flux analysis. One hour prior to the assay, cells were washed and the culture medium was replaced with XF assay medium (modified DMEM, pH 7.4; Seahorse Bioscience), supplemented with glucose (25 mmol/L), glutamine 2 (mmol/L), and sodium pyruvate (2 mmol/L), before being incubated at 37 °C, at atmospheric CO_2_ levels. Oxygen consumption rate (OCR) and extracellular acidification rate (ECAR) were measured using the XF^e^96 analyser (Seahorse Bioscience). Basal OCR measurements were taken followed by injection of the following compounds from the XF Cell Mito Stress Test Kit (Seahorse Bioscience): oligomycin (1 µmol/L, injection port A), FCCP (1 µmol/L, injection port B), and combined antimycin A (0.5 µmol/L), and rotenone (0.5 µmol/L) (injection port C). All data were normalised to protein concentration, measured via BCA assay.

### 4.12 Transmission electron microscopy and mitochondrial measurements

Cells were grown and transfected on plastic coverslips. 72 h post-transfection, cells were washed in PBS, and fixed in warm glutaraldehyde (2.5%), PFA (2%), and PIPES buffer (0.1 M, pH7.2) for 30 min at room temperature. Secondary fixation was carried out for 1 h at 4 °C in osmium tetroxide (1%) in PIPES buffer (0.1 M, pH 7.2), followed by tertiary fixation in uranyl acetate (0.5%) over night at 4 °C. Samples were embedded in resin after ethanol dehydration and gradual infiltration with Agar100 epoxy resin. After heat polymerisation, samples were sectioned (90 nm) using the Leica UC7 ultramicrotome with a diamond knife and post stained with Reynolds’ lead citrate. 5–7 cells per sample were imaged using a FEI Tecnai 12 Transmission Electron Microscope (TEM) at 120 kV, using a Gatan US1000 digital CCD camera. Mitochondria were randomized, counted ‘blinded’ to the user, and the length (by placing a tip-to-tip line across the longest axis of each mitochondrion), and area (by drawing around and selecting each individual mitochondrion) quantified using scale bars and ImagePro Plus 6 (Media Cybernetics) [40], [41].

### 4.13 Mitochondrial DNA content

Mitochondrial DNA relative to nuclear DNA was assessed using real-time PCR and a Bio-Rad CFX96 detection system and iTaq Universal SYBR Green Supermix. DNA was extracted from cell pellets using the Qiagen DNeasy Blood and Tissue kit following the manufacturers protocol. Primers specific to nuclear DNA genes were Actin (*Actb* Fwd-CTGCCTGACGGCCAGG, Rev-GAAAAGAGCCTCAGGGCA) and Succinate dehydrogenase complex subunit A (*Sdha* Fwd-TACTACAGCCCCAAGTCT, Rev-TGGACCCATCTTCTATGC). Primers for mitochondrial DNA genes were cytochrome B (*mtCytb* Fwd- CCACTTCATCTTACCATTTATTATCGC, Rev- TTTTATCTGCATCTGAGTTTAATCCTGT) and subunit II of mitochondrial cytochrome C oxidase (*mtCO2* Fwd-CTACAAGACGCCACAT, Rev- GAGAGGGGAGAGCAAT). In each reaction mixture 2 ng of DNA was used.

### 4.14 Statistical analysis

Data are expressed as mean±SEM. Comparisons between NS and GCH siRNA were made by the unpaired Student's *t*-test. Experiments testing multiple treatments between cell type and knockdown were compared by two-way ANOVA, with post-hoc tests applied to test for significance between knockdown and treatments, as outlined in the figure legends. *P*<0.05 was considered statistically significant.

## Author contributions

JB, AS, RF, BJR, MJC: Carried out experiments and analysed data. BMK, JM, RWM: Provided technical assistance, data interpretation and reviewed the manuscript. KMC and MJC: Conception of the ideas, interpreted the data. JB, AS and MJC: Wrote the manuscript. No conflicts of interest.

## Footnotes

Appendix A Supplementary data associated with this article can be found in the online version at doi:10.1016/j.freeradbiomed.2017.01.012.
