# Supplementary material for: A novel role for endothelial tetrahydrobiopterin in mitochondrial redox balance
Source: Free Radic Biol Med. 2017 Mar;104:214–25. doi: 10.1016/j.freeradbiomed.2017.01.012 (PMC5338462; doi:10.1016/j.freeradbiomed.2017.01.012)
Supplement: Supplementary file 1 — Supplementary material [file mmc1.pdf]

SUPPLEMENTAL FIGURE 1

**Target protein**	**Supplier**	**Dilution**
GTP cyclohyrdolase (GTPCH)	Prof Steve Gross, Cornell	1:50,000
Glyceraldehyde 3-phosphate dehydrogenase (GAPDH)	Millipore	1:10,000
Succinate dehygrogenase (SDH)	Cell Signaling Technology	1:1000
Voltage-dependent anion channel (VDAC)	Cell Signaling Technology	1:1000
Cytochrome c	Cell Signaling Technology	1:1000
Cytochrome c oxidase subunit 4 (COX IV)	Cell Signaling Technology	1:1000
Pyruvate dehydrogenase (PDH)	Cell Signaling Technology	1:1000
TOM20	BD Transduction Laboratories	1:2000
P62	Sigma Aldrich	1:500
Thioredoxin (TRX)	BD Transuction Laboratories	1:5000
Thioredoxin interacting protein (TXNIP)	Sigma Aldrich	1:1000
Glutathione reductase (GR)	Santa Cruz	1:5000
Fumarate hyratase (FH)	Santa Cruz	1:2500
Mitofusin 2	Cell Signaling Technology	1:1000
OPA 1	Cell Signaling Technology	1:1000
